# Exploring spurious penicillin allergy prevalence in general practices in North Wales

**DOI:** 10.1093/jacamr/dlag190

**Published:** 2026-08-27

**Authors:** Clara Tam, Gill Boothman, Alison Hughes, Neil Powell

**Affiliations:** Pharmacy Department, Betsi Cadwaladr University Health Board, Bangor, Wales, UK; Pharmacy and Medicine Management, Betsi Cadwaladr University Health Board, Bangor, Wales, UK; Pharmacy and Medicine Management, Betsi Cadwaladr University Health Board, Bangor, Wales, UK; Pharmacy Department, Royal Cornwall Hospital Trust, Truro, UK; School of Biomedical Sciences, University of Plymouth, Plymouth, UK

Penicillin allergy (PenA) is the most reported allergy in healthcare.^1^ A recent systematic review reported the global prevalence of PenA to be 9.4%, but only 3% of the included studies were from primary care.^2^ Two studies in England reported the prevalence of penA in primary care to be between 6% and 8%.^3,4^ PenA prevalence in Wales is unknown.

People with a PenA label were found to have an increased total number of antibiotic prescribings.^3^ Health outcomes were also worse in those with a PenA label, with increased MRSA infection, *Clostridioides difficile* infection, increased length of hospital stays and mortality.^1,3,5^ However, only 5% reported to have PenA are truly penicillin allergic when tested, and therefore these associated harms are avoidable.^6^ To optimize antibiotic prescribing and to improve antimicrobial stewardship, an accurate PenA record with removal of inaccurate and low-risk PenA records is vital.

The medicine management team in Betsi Cadwaladr University Health Board, North Wales, serving a population of more than 700 000 people, worked collaboratively with the antimicrobial pharmacy team to determine (1) the prevalence of PenA in the local population, (2) the extent of allergy documentation, and (3) the proportion of people who had symptoms in keeping with true PenA allergy (Table 1: levels 2 and 3) documented across general practices in North Wales. This work was conducted through the local enhanced clinical effectiveness scheme between April 2023 and April 2024, where each practice was financially incentivised with 0.085-pound sterling per registered person, on completion and return of the data collection proforma to the medicine management team for analysis. Ethics approval was not required for this service evaluation. GPs were provided with read codes to identify people with PenA to determine the prevalence of PenA. In each participating practice, a proportion of people with PenA were randomly selected to assess the proportion of people who had had the nature of the reaction recorded, and the proportion of people who had had symptoms in keeping with true PenA allergy recorded within the patient summary record. The classification of symptoms in keeping with true allergy was determined by the symptoms recorded on patient summary (see Table 1). Symptoms were adapted from The British Society for Allergy & Clinical Immunology guideline^6^ and CATALYST: challenging antibiotic allergy status.^7^

**Table 1.** Potential symptoms listed in the penicillin allergy records grouped into likely non-allergy (level 1) and allergy (levels 2 and 3) and allergy severity (level 2: low severity, and level 3: high severity)

Intolerance/non-allergic characteristics	Symptoms in keeping with true allergy	
Level 1	Level 2	Level 3
Gastrointestinal symptoms (nausea, vomiting, diarrhoea) Secondary infection (e.g. thrush or *Clostridioides difficile* colitis) Mild symptoms (e.g. headache, joint pain, change of taste in mouth) Family history of penicillin allergy but without personal history of it Mild renal/liver impairment (unless resulting in severe renal failure/impairment)	Diffuse or localized rash with no other symptoms more than 1 h post dose Non-specific childhood rash	Collapse requiring resuscitation Itchy rash/urticaria (hives) within 1 h of dose Blistering/peeling of skin Oral/genital ulceration New wheeze Swelling of lips/tongue/eyes (angioedema) Severe renal/liver impairment/failure (e.g. significant deranged biochemistry or requiring dialysis/transplant)

Of 96 general practices, 93 (97%) responded; these serve a population of 710 418 registered people. We identified 51 845 people who carried a PenA record. The prevalence of PenA in this population was 7.3%. Due to the large number of people with PenA, practices were asked to select a reasonable proportion of people to conduct a review of documentation. Most practices selected 20 people to review documentation on patient summary, but four practices reviewed fewer than 20 patients to conduct this review. In total, 1823 people with a PenA record were included to review: 35% (*n* = 649) had the details of the reaction recorded; 23% (*n* = 423) had symptoms in keeping with true allergy (level 2 or level 3 symptoms) recorded; and 12% (*n* = 226) had symptoms of intolerances or non-allergic characteristics (level 1 symptoms) recorded in patient summary.

In this study, we conclude that PenA prevalence in North Wales is comparable to that reported from other parts of the world. We found there was a low compliance in documentation of the nature of reaction, despite 2014 national guidance recommendations.^8^ Documentation of drug allergy, particularly the nature of reaction, is key to determining whether a reaction is likely on re-exposure to penicillin, and crucial information for clinicians in order to decide whether penicillin is the optimal treatment choice. Yet, less than half of the records had the nature of reaction recorded in the patient summary. This is partly due to the configuration of the electronic clinical system used in general practice, where nature of reaction is not a mandatory field when recording an allergy. This could also be attributable to the lack of knowledge of PenA by the public and healthcare professionals, but this was not assessed as part of this study. Furthermore, those who had intolerances or non-allergy characteristics from previous penicillin exposure would benefit from direct de-labelling with the appropriate guidance. Finally, within the cohort of people who had symptoms in keeping with true PenA symptoms (levels 2 and 3), we estimate that a significant proportion of people with low-risk (level 2) symptoms are not allergic to penicillin after formal PenA testing.^9^ This could be a significant patient safety issue if it is not appropriately addressed.

These findings demonstrate a need to improve accuracy of allergy documentation. This is a call for national bodies in Wales to develop national guidelines and actions to support healthcare professionals to address this patient issue; along with education of general practices and feedback to companies supplying the clinical health systems. In addition, we have identified a need for a robust PenA de-labelling service for the people in North Wales in order to safely remove low-risk PenA records to ensure people receive the most appropriate antibiotics when needed, optimizing treatment outcomes and avoiding unintentional harms.
